# Delta external fixation as a suitable option for definitive fixation of osteoporotic pilon fractures in biomechanics: a finite element analysis

**DOI:** 10.3389/fbioe.2026.1719193

**Published:** 2026-05-07

**Authors:** Ping Chen, Luyuan Chen, An Hu, Wen Zhao, Qinglan Xu, Wenhan Liu, Wenhui Zhang, Ji Qi, HuanHuan Gao, Xiubing Yu, Yili Chen, Haizhou Wang

**Affiliations:** 1 Guangdong Provincial Hospital of Traditional Chinese Medicine, Guangzhou University of Chinese Medicine, Guangzhou, Guangdong, China; 2 Stomatology Center, Shenzhen Hospital, Southern Medical University, Shenzhen, Guangdong, China; 3 School of Instrumentation and Optoelectronic Engineering, Beihang University, Beijing, China; 4 Department of Neurology and Psychology, The Fourth Clinical Medical College of Guangzhou University of Chinese Medicine, Shenzhen, Guangdong, China; 5 Hong Kong Baptist University, Kowloon Tong, Hong Kong, China

**Keywords:** biomechanical stability, external fixation, finite element analysis, osteoporosis, pilon fracture

## Abstract

**Introduction:**

Pilon fractures are high-energy traumatic injuries characterized by complex metaphyseal fractures that involve the distal tibial articular surface, often accompanied by severe soft tissue damage. External fixation represent a crucial treatment treatment modality, particularly for elderly patients with osteoporosis (OP). This study aimed to identify the most suitable external fixation strategy for OP-associated Pilon fractures by comparing the biomechanics of three common used external fixations strategy.

**Methods:**

A finite element model was developed to simulate Ruedi-Allgower III/AO43C Pilon fractures combined with fibular fractures. This model was used to simulate gait cycles (swing and stance phases) for analysis of the key biomechanical parameters, including maximum von Mises stress (Max_VMS) in both bony structures and external fixation, as well as micromovements of fracture fragments, distal tibial fragments, the fibula, and the tibio-talar articular surface. These analyses were performed under both normal and OP bone conditions.

**Results:**

For the same external fixation and phase, the Max_VMS in bony structures was higher in normal bone than in OP bone, whereas the Max_VMS in the external fixation was significantly higher under OP conditions. Across all fixation, OP bone consistently exhibited greater fracture fragment micromovement than normal bone. Among the three fixations evaluated, the Delta external fixation showed the lowest Max_VMS on the tibio-talar articular surface (1.87 MPa in OP during the swing phase; <1.83 MPa in OP during the stance phase) and the minimal fragment micromovement. Specifically, during the swing phase, the Delta fixation restricted fragment micromovement to 0.15–0.40 mm (the optimal range for fracture healing) in both bone conditions, compared to 0.40–2.00 mm range observed with Unilateral and Ilizarov fixations. During the stance phase, Delta fixatior maintained fragment micromovement below 2 mm (the threshold for fixation failure) in both bone conditions, a threshold that the other two fixation methods exceeded. Additionally, the Delta fixation prevented excessive stress concentration on lateral distal tibial fragments and reduced anterior fragment micromovement via K-wires in the first phalanx.

**Discussion:**

Among the three external fixation simulation, The finite element analysis result shows the Delta external fixation have superior biomechanical stability—characterized by reduced stress concentration and optimal fragment micromovement—for Pilon fractures fixation, particularly in OP. Its ability to restrict micromovement facilitates healing during the swing phase, while keeping micromovement below the threshold for fixation failure during the stance phase establishes it as the preferred fixation method for OP-associated Pilon fractures, which may promote early ambulation and fracture healing.

## 1 Introduction

Pilon fractures are metaphyseal fractures involving the articular surface of the distal tibia. It is primarily caused by high-energy axial loading (Topliss et al., 2005) and accounts for 5%–7% of all tibial fractures (Mauffrey et al., 2011). Given the complexity of these injuries, they are often accompanied by severe soft tissue damage. Open reduction and internal fixation (ORIF) is commonly used in the treatment of Pilon fractures. However, optimalsurgery timing and fixation strategy selection remain controversial (Kottmeier et al., 2018). This controversy is largely attributable to the high complication rate associated with internal fixation (Ketz and Sanders, 2012). When ORIF is not appropriate as primary treatment for Pilon fractures, external fixation serves as an effective method for temporary stabilization (Lavini et al., 2014). The utilization of external fixation can not only control the first-stage damage, but also provide safe and effective soft tissue conditions for the treatment of secondary internal fixation. This approach avoids further soft tissue trauma caused by emergency incisions, significantly reduces the postoperative infection rate, and leads to satisfactory clinical outcomes.

External fixation has been reported to exhibit satisfactory efficacy as a terminal fixation modality for Pilon fractures (Nozaka et al., 2020; Scaglione et al., 2018; Galante et al., 2016), highlighting advantages such as procedural simplicity, shorter average hospital stays, lower cost, and feasibility in emergency treatment. However, the primary disadvantages of external fixation for Pilon fractures include the following: (1) External fixation may lead to ankle stiffness and chondrodystrophy, and may increase the incidence of complex regional pain syndrome. (2) Hematoma organization at the fracture site and contracture of the surrounding soft tissue, increasing the difficulty of subsequent reduction and the risk of non-union (Nielsen et al., 2018). Nozaka et al. (2020) applied the Ilizarov ring external fixation to treat elderly patients with Pilon fractures. And there was no statistically significant difference in postoperative ankle joint mobility and functional scores compared to patients who underwent internal fixation. However, the external fixation group demonstrated significantly shorter hospital stays and lower complication rates. These findings suggest that external fixation as terminal treatment for OP Pilon fractures may facilitate earlier weight-bearing and functional recovery. Contemporary clinical evidence further support the use of hybrid, ring and Ilizarov external fixations for terminal fixation of Pilon fractures. These external fixations are recognized for their stable fixation properties, which can facilitate patients to early ambulation, and maintain the mobility of the foot and ankle, allowing to walk independently in later stage (Nozaka et al., 2020; Galante et al., 2016; Harada et al., 2019). Collectively, this evidence confirms external fixation as a highly viable treatment option for Pilon fractures.

Osteoporosis (OP) is a prevalent bone disorder among the elderly. Approximately 50% of women and 20% of men over the age of 50 may experience osteoporosis fracture (Wang et al., 2021; Siris et al., 2014). In elderly fracture patients with OP, poor stability of internal fixation during treatment are not conducive to fracture healing. Prolonged bed rest may exacerbate disuse osteoporosis and increase the risk of further fractures (Rolvien and Amling, 2022). Thus, the use of external fixation as a terminal fixation treatment for osteoporotic Pilon fracture can enable earlier weight-bearing and functional recovery as early as possible and gain benefits. And few studies have investigated the stability of external fixation in the management of Pilon fractures. Muhammad et al. conducted a finite element analysis (FEA) of six Delta external fixation configurations for Pilon fractures, and identified the most suitable and stable triangular fixation configuration (Ramlee et al., 2018). Meanwhile, they simulated the weight-bearing condition of this most stable configuration under OP bone conditions (Ramlee et al., 2020). The above studies provide valuable biomechanically insights of the Delta external fixation for Pilon fractures.

Nevertheless, clinical applications of this specific configuration remains limited, and mechanical analyses of other external fixators used in clinical practice are lacking, particularly in the OP. Additionally, Nozaka et al. (2020) reported favorable outcomes with Ilizarov ring external fixation for elderly patients with Pilon fractures, its biomechanical performance in this population remains unclear.

Currently, there is limited research on the external fixation strategy for Pilon fractures with OP. Our study aimed to simulate and compare the stability of different external fixation strategy for Pilon fractures under the condition of OP. In this study, the maximum stress, associated fracture fragment micro-micromovement, external fixation and bone micromovement of three kinds of external fixations (Unilateral, Delta, and Ilizarov) during gait cycles under two different bone conditions (normal and OP) were compared. The primary objective was to identify the most appropriate external fixation strategy for osteoporotic Pilon fractures.

## 2 Materials and methods

### 2.1 Finite element (FE) fracture modeling

#### 2.1.1 Data source and preprocessing

Utilized existing computed tomography (CT) datasets to reconstruct 3D ankle models (20 bones including tibia, fibula, talus, etc.) via Mimics software (Materialise, Leuven, Belgium).

Post-reconstruction: Surface smoothing and meshing were performed using Geomagic Studio software (3D Systems, United States of America) to ensure model accuracy.

#### 2.1.2 Pilon fractures model construction

**FIGURE 1:**
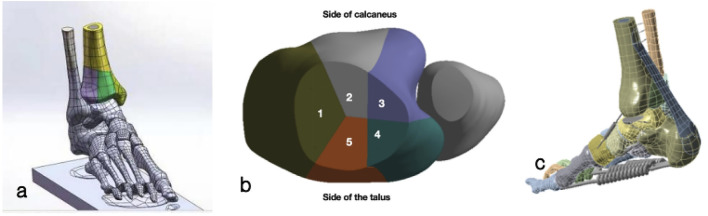
Schematic of the finite element (FE) model for Pilon fractures **(a)** Overall model: A 3D FE model of Ruedi-Allgower III/AO43C Pilon fracture combined with fibular fracture, reconstructed from clinical CT images and including 20 bones (tibia, fibula, talus, calcaneus, etc.) and 14 major ligament/soft tissue structures. **(b)** Fracture fragments: Visualization of the Y-shaped distal tibial fracture fragments (consistent with clinical imaging data) and associated fibular fracture (located 5–10 cm above the tibiofibular joint surface). **(c)** Ligament schematic: Schematic representation of ligaments (interosseous membrane, Achilles tendon, plantar fascia, etc.) simulated using spring elements, with anatomical attachments based on the Knight Color Atlas of Human Anatomy.

Ruedi-Allgower III and AO43C Pilon fractures were selected as the study models (Marsh et al., 2007), as these high-energy injuries are often associated with significant soft tissue damage and are more suitable for initial stabilization with external fixation (Lavini et al., 2014). According to literature (Ruedi and Allgower, 1969), approximately 90% of AO43C and Ruedi Allgower type III Pilon fractures presnt with a Y-shaped fracture lines. Furthermore, it has been reported that Pilon fracture with high-energy injury was frequntly accompanied by fibula fractures, typically located 5–10 cm above the tibiofibular joint surface (Cole et al., 2013). Meanwhile the fracture line patterns of Pilon fracture model in this study was were modeled based on the clinical imaging data described by Chen (2022). SolidWorks 2018 (Dassault Systèmes, France) was used for osteotomy modeling. The Pilon fracture (Ruedi-Allgower III and AO43C) combined with fibula fracture model was successfully constructed (Figure 1).

#### 2.1.3 Boundary and loading conditions

Following model assembly, the complete construct was imported into ANSYS 19.0 (Ansys, Inc., United States of America) for meshing. The material properties assigned to the mesh, including bone density and cartilage parameters, were adopted from established references (Busel and Watson, 2017; Alonso et al., 2017; Morales et al., 2017; Kim H. J. et al., 2011; Sun et al., 2008), as detailed in Table 1. Accounting for bone thickness would result in markedly different interfacial contact conditions between K-wires and bone relative to those in the normal model. Thus, the material properties of bone in the OP model were adjusted by excluding the influence of bone thickness to enable valid comparison with the normal group, thereby yielding more robust and persuasive conclusions.

**TABLE 1:** The model attribute setting.

Bone model	Material	Material property	Grid method	Grid size (mm)	Density (kg/m^3^)	Young’s modulus (MPa)	Poisson’s ratio	References
Normal	Cortical bone	Solid, linear elastic material	tetrahedron	4.5	1980	7,300	0.3	Alonso et al. (2017), Morales et al. (2017), Kim H. J. et al. (2011), Sun et al. (2008)
	Cancellous bone	Solid, linear elastic material	tetrahedron	4.5	430	1,100	0.3	Alonso et al. (2017), Morales et al. (2017), Kim H. J. et al. (2011), Sun et al. (2008)
Osteoporosis	Cortical bone	Solid, linear elastic material	tetrahedron	4.5	1980	400	0.3	Alonso et al. (2017), Morales et al. (2017), Kim H. J. et al. (2011), Sun et al. (2008)
	Cancellous bone	Solid, linear elastic material	tetrahedron	4.5	430	61	0.3	Alonso et al. (2017), Morales et al. (2017), Kim H. J. et al. (2011), Sun et al. (2008)
​	Cartilage	Solid, linear elastic material	tetrahedron	1.2	1,300	1	0.4	Alonso et al. (2017), Morales et al. (2017), Kim H. J. et al. (2011), Sun et al. (2008)

A total of 14 major ligaments and soft tissue structures including the interosseous membrane, Achilles tendon, plantar fascia, and various interosseous ligaments were incorporated into the model. Their anatomical attachments were defined based on descriptions in the Knight Color Atlas of Human Anatomy. These structures were simulated using spring elements. The material properties for the ligaments were derived from the literature (Beumer et al., 2003; Siegler et al., 1988; Liacouras and Wayne, 2007; Nie et al., 2017; Iaquinto and Wayne, 2010; Kai et al., 2009; Liu et al., 2018) and are summarized in Table 2.

**TABLE 2:** The stiffness of each ligament.

Ligament	Commissura	Stiffness	References
Interosseous membrane (4)	Tibia -- Fibula	400	Beumer et al. (2003)
Anterior tibiofibular ligament	Tibia -- Fibula	78	Siegler et al. (1988)
Posterior tibiofibular ligament	Tibia -- Fibula	101	Liacouras and Wayne (2007)
Anterior tibiotalar ligament	Tibia–Talus	70	Siegler et al. (1988)
Posterior tibiotalar ligament	Tibia–Talus	80	Siegler et al. (1988)
Calcaneofibular ligament	Calcaneus–fibula	70	Siegler et al. (1988)
Calcaneal tibial ligament	Calcaneus–scaphoid bone	122	Siegler et al. (1988)
Tibioscaphoid ligament	Tibia–scaphoid bone	40	Siegler et al. (1988)
Calcaneal scaphoid ligament	Calcaneus–scaphoid bone	70	Siegler et al. (1988), Nie et al. (2017)
Anterior talofibular ligament	Talus–fibula	89	Iaquinto and Wayne (2010)
Posterior talofibular ligament	Talus–fibula	775	Iaquinto and Wayne (2010)
Talonavicular ligament	Talus–scaphoid bone	70	Kai et al. (2009)
Medial plantar aponeurosis	Metatarsal bone–Calcaneus	200	Kai et al. (2009)
Intermediate plantar aponeurosis	Metatarsal bone–Calcaneus	230	Kai et al. (2009)
The lateral plantar aponeurosis	Metatarsal bone–Calcaneus	180	Kai et al. (2009)
Achilles tendon	Tibia-Calcaneus	11.43	Liu et al. (2018)

### 2.2 Model validation

**FIGURE 2:**
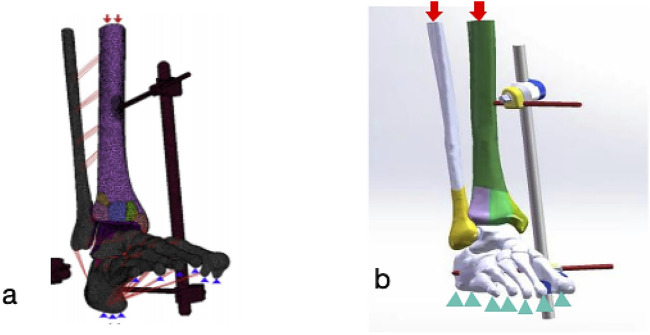
Loading conditions and external fixator constraints **(a)** Delta external fixation configuration and loading setup adapted from Ramlee et al. (2014); **(b)** External fixator configuration and loading setup used in the present study. Note: Blue triangles indicate fixed constraints applied to the metatarsal and calcaneal bones; red arrows denote vertical loads (70 N for swing phase, 350 N for stance phase) applied to the proximal cross-sections of the tibia and fibula, simulating gait cycles of a 70 kg individual.

The model in normal bone mineral density was utilized for simulation and verification, and the Unilateral external fixation configuration, as discribed by Ramlee et al. (Ramlee et al., 2014) (Figure 2a), was applied to the model. A frictional contact interface was defined between the fixator and the bone. The Young’s modulus and friction coefficient of the external fixation consistent with the values reported in the literature (Figure 2b). Subsequently, the resulting stress distributions in the tibia and calcaneus, as well as the stress and micromovement of the external fixator, were computed and compared against the corresponding data from Reference (Ramlee et al., 2014).

The result demonstrated a strong agreement between the FE model and experimentally measured data reported in the literature (Liacouras and Wayne, 2007). Specifically, the maximum von Mises stress values observed in the tibia and talus were consistent with the previously published results, as detailed in Table 3.

**TABLE 3:** A comparison of experimental results under various conditions.

Loading stress	70 N		350 N	
	Reference	Model	Reference	Model
Calcaneus stress (MPa)	24.9	26.18	130.3	152.09
Tibial stress (MPa)	7.9	12.13	41.5	99.57
External fixation stress (MPa)				
Proximal tibia	25.0	25.58	120.2	112.54
Distal tibia	27.0	33.81	173.2	123.44
calcaneus	45.6	42.02	266.7	109

### 2.3 Sensitivity analysis

Grid density sensitivity analysis was conducted in this non-fracture model without muscle. The differences in stress values among three different grid sizes (3/4.5/6 mm) within the same region were all less than 5%. Thus, the model in this study was considered validated.

### 2.4 External fixator configurations

**FIGURE 3:**
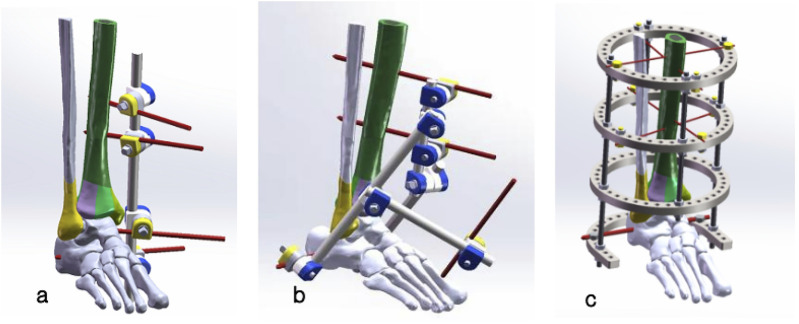
Three external fixation configurations for Pilon fracture treatment **(a)** Unilateral external fixator: Composed of 1×φ11 mm unilateral rod, 4 pin clamps, and 4×φ5 mm K-wires (2 inserted into proximal tibia, 1 into calcaneus, 1 into talus), connected to the main rod for traction and stabilization. **(b)** Delta external fixator: Triangular configuration with multiple φ11 mm short rods, 2×φ5 mm threaded half-pins (proximal tibia), and 2×φ5 mm K-wires (1st metatarsal, calcaneus), forming stable triangular assemblies to restrict fragment displacement. **(c)** Ilizarov external fixator: Ring-based fixator with 3 full rings, 1 half-ring, 4×φ10 mm threaded rods, and 5×φ2/5 mm K-wires (proximal/distal tibia, calcaneus), interconnected to enhance fixation rigidity.

Three configurations were modeled via SolidWorks 2018 and integrated with the fracture model (Figure 3):

1. Unilateral: 1×φ11 mm main rod +4×φ5 mm K-wires (proximal tibia, calcaneus, talus) + 4 pin clamps (Ramlee et al., 2014; Zhang, 2017) (Figure 3a). Two K-wires were inserted perpendicularly into the anteromedial aspect of the proximal tibia, engaging both cortices. A third K-wire was inserted horizontally through the center of the calcaneus, and a fourth K-wire was placed horizontally into the center of the talus body. All K-wires were connected to the main rod via pin clamps to provide traction and stabilization.
2. Delta: Multiple φ11 mm short rods +2×φ5 mm threaded half-pins (proximal tibia) + 2×φ5 mm K-wires (first metatarsal, calcaneus), assembled into a triangular structure (Ramlee et al., 2020) (Figure 3b). The two threaded half-pins were inserted into the proximal tibia in the sagittal plane, engaging both cortices. One K-wire was driven vertically from dorsal to plantar through the first metatarsal, while another K-wire was passed horizontally through the calcaneus. A short rod was fixed to each end of the calcaneal K-wire. The two tibial half-pins were fixed to a separate short rod, aligned parallel to the mechanical axis of the tibia. These three short rod assemblies were then connected by pin clamps to form a stable triangular configuration. The first metatarsal K-wire was fixed to a posterior short rod, which was linked to the lateral short rod of the calcaneal K-wire, thereby stabilizing the forefoot.

Ilizarov: 3 full rings +1 half-ring (5 mm thickness) + 4×φ10 mm threaded rods +5×φ2/5 mm K-wires (proximal/distal tibia, calcaneus) (Nozaka et al., 2020) (Figure 3C). Two φ2 mm K-wires were inserted into the proximal tibia in the sagittal and coronal planes, respectively, and secured to a ring with nuts. Two additional φ2 mm K-wires were inserted into the distal tibia, proximal to the fracture line, using the same technique and fixed to a second ring. The φ5 mm K-wire was inserted horizontally through the center of the calcaneus and secured to the half-ring with nuts. The two proximal rings and the distal half-ring were interconnected by four threaded rods, positioned at the anterior and posterior quadrants of the rings. An additional ring was assembled at the level of the fracture plane for enhanced stability.

Following virtual assembly, each complete fixator construct was imported into ANSYS 19.0. Each configuration was modeled as a single, integrated part with homogeneous material properties. The fixators were defined as solid, linear, and isotropic elastic materials with a Young’s modulus of 110,000 MPa and a Poisson’s ratio of 0.3 (Ramlee et al., 2020). Each model was then meshed; the resulting mesh size and node counts for each configuration are provided in Table 4.

**TABLE 4:** The model attribute setting.

External fixation configuration	Grid method	Grid sixe (mm)	Grid node	Grid element
Unilateral external fixation	tetrahedron	3	371,575	225,751
Delta external fixation	tetrahedron	3	801,860	493,565
Ilizarov external fixation	tetrahedron	3	520,746	185,142

The contact interfaces between fracture fragments and between the K-wires and bone were defined as frictional, with coefficients of 0.3 and 0.4, respectively (Ramlee et al., 2018). The metatarsal and calcaneal bones were assigned fixed constraints. To simulate the swing and stance phases of the gait cycle for a 70 kg individual, vertical loads of 70 N and 350 N were applied to the proximal cross-sections of the tibia and fibula, respectively, based on established biomechanical data (Kim S. H. et al., 2011; Simkin et al., 1985). Under these two loading conditions, simulations were performed for both normal and osteoporotic bone models. The following parameters were computed: the von Mises stress in the tibia, calcaneus, metatarsals, and external fixators; the micromovement of the external fixators; and the stress and micromovement at the fracture fragments.

## 3 Results

### 3.1 Stress distribution

#### 3.1.1 Maximum von mises stress (Max_VMS) distribution in bone

**FIGURE 4:**
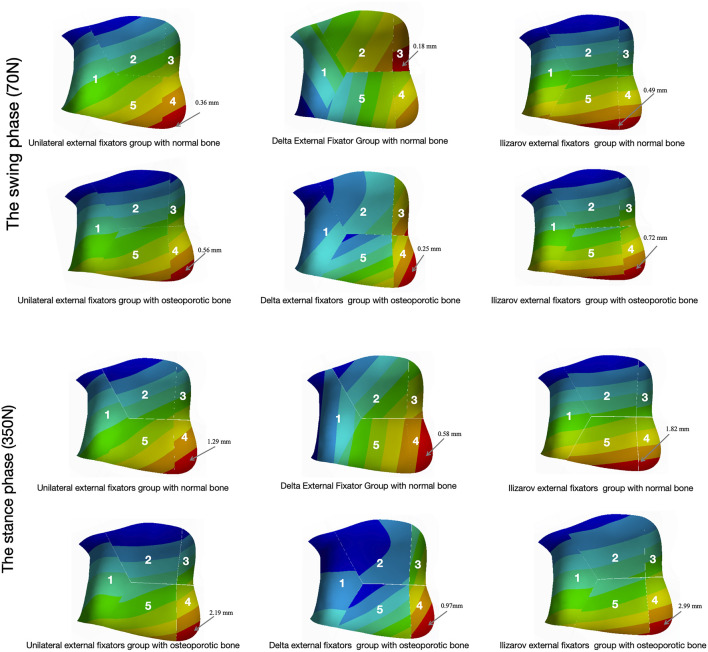
Max_VMS distribution of bone and external fixators Max_VMS of bone tissue (tibia, calcaneus, metatarsals, and fracture fragments) and external fixators (rods, K-wires, pins) under two bone conditions (normal bone, OP bone) during the swing phase (70 N load) and stance phase (350 N load). Stress concentration regions (e.g., bone-pin interface, K-wires) are highlighted.

Overall, the Max_VMS in the bone was consistently concentrated at the bone-pin interface across all configurations and conditions (Figure 4).

During the swing phase: (1) For the Unilateral external fixator in normal bone, the Max_VMS was 6.96 MPa, located at the proximal tibial pin tract. Under osteoporotic conditions, the Max_VMS decreased to 1.98 MPa and was concentrated at the entrance of the calcaneal pin tract. (2) For the Delta external fixator in normal bone, the Max_VMS was 37.54 MPa, concentrated at the distal tibial pin tract. In the osteoporotic model, the Max_VMS was 2.25 MPa, also located at the entrance of the distal tibial pin tract. (3) For the Ilizarov external fixator in normal bone, the Max_VMS was 118.78 MPa, concentrated at the proximal tibial pin tract. Under OP conditions, the Max_VMS was 10.30 MPa, with the stress concentration remaining in the proximal tibial pin tract.

During the stance phase: (1) For the Unilateral external fixator in normal bone, the Max_VMS was 32.62 MPa, concentrated at the calcaneal pin tract. In the osteoporotic model, the Max_VMS was 9.95 MPa and shifted to the proximal tibial pin tract. (2) For the Delta external fixator in normal bone, the Max_VMS was 35.88 MPa, concentrated at the distal tibial pin tract. In the osteoporotic model, the Max_VMS was 9.44 MPa, maintaining its concentration at the distal tibial pin tract. (3) For the Ilizarov external fixator in normal bone, the Max_VMS was 156.10 MPa, concentrated at the proximal tibial pin tract. In the osteoporotic model, the Max_VMS was 13.59 MPa, and similarly concentrated at the proximal tibial pin tract.

#### 3.1.2 Stress distribution in the external fixator

During the swing phase simulation: (1) For the Unilateral external fixator in normal bone, the Max_VMS was 58.68 MPa, with stress concentrated on the K-wire engaging the talus. Under osteoporotic conditions, the Max_VMS increased to 83.10 MPa, also concentrated on the talar K-wire. (2) For the Delta external fixator in normal bone, the Max_VMS was 55.84 MPa, located on the K-wire in the distal tibia. In the osteoporotic model, the Max_VMS was 64.89 MPa, and the stress concentration shifted to the K-wire in the proximal tibia. (3) For the Ilizarov external fixator in normal bone, the Max_VMS was 93.44 MPa, concentrated on the K-wire that secured the fixation ring to the proximal tibia. Under osteoporotic bone conditions, the Max_VMS was 63.33 MPa, with stress similarly concentrated on the proximal tibial K-wire connecting to the fixation ring.

During the stance phase simulation: (1) For the Unilateral external fixator in normal bone, the Max_VMS was 266.48 MPa, with stress concentrated on the talar K-wire. In the osteoporotic model, the Max_VMS rose to 375.83 MPa, remaining concentrated on the same K-wire. (2) For the Delta external fixator in normal bone, the Max_VMS was 174.24 MPa, concentrated on the K-wire in the proximal tibia. In the osteoporotic model, the Max_VMS increased to 265.87 MPa, with stress consistently concentrated on the proximal tibial K-wire. (3) For the Ilizarov external fixator in normal bone, the Max_VMS was 123.05 MPa, located on the K-wire connecting the fixation ring to the proximal tibia. In the osteoporotic model, the Max_VMS was 284.29 MPa, and the stress remained concentrated on this proximal tibial K-wire.

#### 3.1.3 Max_VMS at the distal tibial fracture fragments, fibula, and tibiotalar articular surface

The Max_VMS values for each distal tibial fracture fragment, the fibula, and the tibiotalar articular surface under the two bone conditions (osteoporotic and normal) during the swing phase are summarized in Table 5. The corresponding Max_VMS distributions during the stance phase are presented in Table 6.

**TABLE 5:** The Max_VMS and the maximum micromovement of the each distal tibial fracture fragments, fibula and tibial talar articular surface under both normal and osteoporotic bone conditions during the swing phase.

Result	Unilateral				Delta				Ilizarov			
	VMS (Mpa)		micromovement (mm)		VMS (Mpa)		micromovement (mm)		VMS (Mpa)		micromovement (mm)	
	Normal	OP	Normal	OP	Normal	OP	Normal	OP	Normal	OP	Normal	OP
Fragment1	4.24	0.71	0.42	0.69	0.25	0.13	0.17	0.23	9.06	1.97	0.70	1.05
Fragment2	4.76	0.44	0.45	0.72	1.01	0.35	0.17	0.25	2.38	0.36	0.65	1.02
Fragment3	1.25	0.22	0.49	0.82	0.41	0.22	0.18	0.29	3.43	0.64	0.69	1.15
Fragment4	0.92	0.23	0.50	0.82	0.92	0.17	0.18	0.29	1.31	0.42	0.83	1.20
Fragment5	0.64	0.46	0.48	0.80	1.19	0.30	0.17	0.27	2.20	0.97	0.82	1.25
Fibia	0.83	0.07	0.50	0.59	0.04	0.02	0.14	0.18	3.32e-6	1.09e-3	0.25	0.55
tibial talar joint	4.24	0.71	0.36	0.56	0.25	0.22	0.17	0.25	9.05	1.97	0.49	0.72

**TABLE 6:** The Max_VMS and the maximum micromovement of the each distal tibial fracture fragments, fibula and tibial talar articular surface under both normal and osteoporotic bone conditions during the stance phase.

Result	Unilateral				Delta				Ilizarov			
	VMS (Mpa)		micromovement (mm)		VMS (Mpa)		micromovement (mm)		VMS (Mpa)		micromovement (mm)	
	Normal	OP	Normal	OP	Normal	OP	Normal	OP	Normal	OP	Normal	OP
Fragment1	10.25	1.90	1.49	2.61	2.95	1.29	0.52	0.86	67.40	10.08	2.67	
Fragment2	4.72	4.92	1.59	2.73	4.18	2.43	0.56	0.92	6.90	3.92	2.51	4.12
Fragment3	3.28	1.30	1.78	3.10	1.44	1.87	0.59	1.13	6.60	5.03	2.68	3.83
Fragment4	5.04	3.12	1.80	3.18	1.11	1.46	0.60	1.09	7.42	4.51	3.15	4.58
Fragment5	4.79	4.21	1.74	3.07	1.48	1.85	0.56	1.00	6.65	4.92	3.16	5.04
Fibia	1.82	0.13	1.27	2.20	1.38	0.20	0.43	0.62	1.74e-05	2.91e-03	0.94	5.08
tibial talar joint	10.08	4.92	1.29	2.19	2.95	1.87	0.58	0.97	67.40	10.08	1.82	2.18

### 3.2 Micromovement distribution

#### 3.2.1 Overall micromovement

**FIGURE 5:**
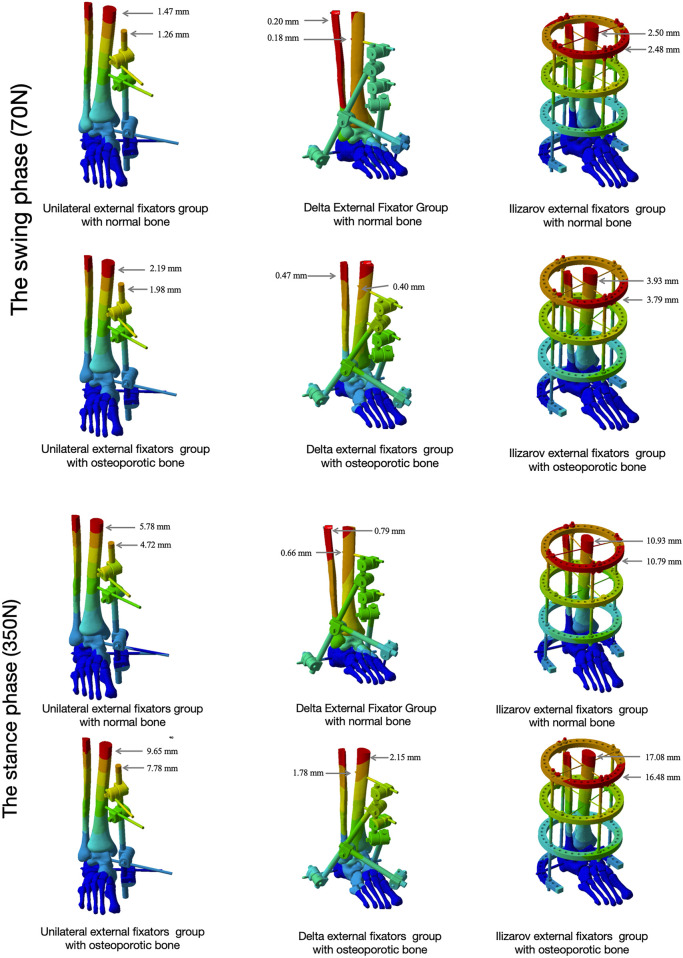
Maximum micromovement of bone and external fixators Maximum micromovement (displacement) of bone tissue (proximal tibia, fracture fragments, fibula) and external fixators (rods, K-wires) under two bone conditions (normal bone, OP bone) during the swing phase (70 N load) and stance phase (350 N load). Data reflect the overall stability of the fixator-bone construct during gait cycles.

As the load was applied proximally to the tibia and fibula, the maximum micromovement occurred at the proximal tibia across all fixator types and bone conditions, with the exception of the Delta external fixator in normal bone (Figure 5).

**FIGURE 6:**
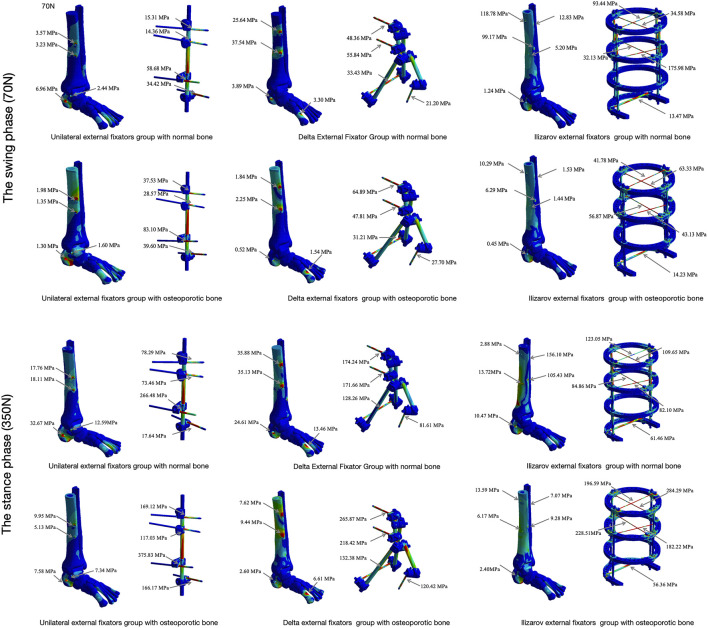
Maximum micromovement of the tibio-talar articular surface Maximum micromovement (displacement) of the tibio-talar articular surface under two bone conditions (normal bone, OP bone) during the swing phase (70 N load) and stance phase (350 N load), comparing the three external fixation configurations (Unilateral, Delta, Ilizarov). Micromovement values are presented for key articular fragments (e.g., anterolateral fragment 4, posterolateral fragment 3).

During the swing phase: (1) The maximum bone micromovement for the Unilateral external fixator was 1.26 mm in normal bone and 1.98 mm in the osteoporotic model. (2) The maximum bone micromovement for the Delta external fixator was 0.18 mm in normal bone and 0.40 mm in the osteoporotic model. (3) The maximum bone micromovement for the Ilizarov external fixator was 2.48 mm in normal bone and 3.79 mm in the osteoporotic model. (4) The maximum fracture fragment micromovement for the Unilateral external fixator was 1.47 mm in normal bone and 2.19 mm in the osteoporotic model. (5) The maximum fracture fragment micromovement for the Delta external fixator was 0.20 mm in normal bone and 0.47 mm in the osteoporotic model. (6) The maximum fracture fragment micromovement for the Ilizarov external fixator was 2.50 mm in normal bone and 3.93 mm in the osteoporotic model. (7) The detailed micromovements of the distal tibial fracture fragments, fibula, and tibiotalar joint surface are provided in Figure 6; Table 5.

During the stance phase: (1) The maximum bone micromovement for the Unilateral external fixator was 4.72 mm in normal bone and 7.78 mm in the osteoporotic model. (2) The maximum bone micromovement for the Delta external fixator was 0.66 mm in normal bone and 1.78 mm in the osteoporotic model. (3) The maximum bone micromovement for the Ilizarov external fixator was 10.79 mm in normal bone and 16.48 mm in the osteoporotic model. (4) The maximum fracture fragment micromovement for the Unilateral external fixator was 5.78 mm in normal bone and 9.65 mm in the osteoporotic model. (5) The maximum fracture fragment micromovement for the Delta external fixator was 0.79 mm in normal bone and 2.15 mm in the osteoporotic model. (6) The maximum fracture fragment micromovement for the Ilizarov external fixator was 10.93 mm in normal bone and 17.08 mm in the osteoporotic model. (7) The detailed micromovements of the distal tibial fracture fragments, fibula, and tibiotalar joint surface are provided in Figure 6; Table 6.

## 4 Disscussion

Pilon fractures are complex, high-energy injuries. Despite ongoing advancements in radiological evaluation, there is still no universally accepted classification system for these fractures. Although the Ruedi-Allgower classification has been recognized as one of the most reliable systems, its inter-observer consistency remains relatively low (Ruedi and Allgower, 1969). This lack of consensus poses a significant challenge for FE modeling, impeding the accurate simulation of clinically representative Pilon fracture patterns. Many previous FE studies have utilized simplified distal tibial osteotomy models (Pirolo et al., 2015) or multiple articular surface osteotomies (Ramlee et al., 2014), which lack a foundation in specific clinical fracture data. Without reference to clinical imaging measurements, such models may fail to accurately represent the complex reality of Pilon fractures. To address this limitation, the present study adopted a Pilon fracture model developed by Chen (2022), which was constructed based on detailed clinical imaging measurements. Validation results indicated that this model possesses reasonable reliability. Consequently, the use of this clinically derived fracture model in our study may enhance the clinical relevance and significance of the findings.

Previous studies have suggested that definitive external fixation can be highly beneficial for patients with Pilon fractures (Nozaka et al., 2020; Scaglione et al., 2018; Galante et al., 2016). This approach provides stable yet flexible fixation, facilitating early ankle mobilization (Babis et al., 2010; Seibert et al., 2010). But in the early stage of rehabilitation, the range of flexion and extension of the stabilized ankle joint is limited because all fractuce fragment need fixed in an actual clinical treatment. During this period, when the fracture stabilized by external fixation, the sole of the foot bears vertical pressure in contact with the ground during walking. Thus, the simulation of vertical forces and rigid constraints can effectively simulate the mechanical environment during the most realistic rehabilitation exercises.

Furthermore, the clinical importance of early mobility is further emphasized by the adverse effects of prolonged bed rest on bone density—an issue of particular concern in osteoporotic patients. As previously reported (Nie et al., 2017), individuals with severe lower limb fractures can lose up to 50% of their tibial cancellous bone mass after just 1 week of non-weight-bearing, with recovery of this bone mass requiring twice the duration of the immobilization period. Therefore, achieving early weight-bearing post-surgery is crucial for OP patients to mitigate bone loss. Furthermore, elderly patients often present with multiple comorbidities and may require treatment with oral anticoagulants, which frequently delays ORIF. Such delays prolong perioperative immobility and increases the risk of complications associated with prolonged bed rest. In contrast, closed reduction and external fixation is less invasive, minimizes soft tissue disruption, promotes vascularization at the fracture site, and thereby enhances fracture healing. These characteristics make it particularly suitable for elderly patients with fragile soft tissues (Chandran et al., 2006).

Additionally, Potter et al. noted that external fixation for Pilon fractures could be performed under local anesthesia. They also reported that the use of two free loops for traction could alleviate pressure from the gastrocnemius muscle, thereby reducing preoperative dislocation and facilitating closed reduction (Potter et al., 2019). However, a notable limitation of external fixation is the high incidence of pin tract infection and malunion associated with improper K-wire placement (Beumer et al., 2003; Siegler et al., 1988). This complication is typically attributed to high stress concentrations resulting from unstable fracture fixation. Despite growing interest in external fixation as a definitive treatment for Pilon fractures, few studies have thoroughly investigated its underlying mechanical rationale, particularly in the context of osteoporotic bone.

Therefore, when considering definitive external fixation for Pilon fractures in OP patients, it is critical to evaluate the mechanical environment of the fixator-bone construct during post-treatment, standing, and rehabilitation. This assessment is essential for determining the suitability of a specific external fixator configuration for definitive fixation in both normal and osteoporotic bone. Our results demonstrated that the bone stress in normal bone models was higher than in OP bone models under all external fixators. This finding is likely attributable to the superior material strength of normal bone. Conversely, the stress on the external fixator itself was substantially higher in OP bone models than in normal bone models for the same fixator configuration. This indicates that in OP patients, a greater proportion of the mechanical load is transferred to and concentrated on the fixator during standing and walking. Consequently, achieving stable fixation in osteoporotic bone is more dependent on the inherent mechanical stability of the external fixator structure itself.

A high incidence of pin tract infection and fracture malunion has been associated with improper selection of K-wire position and external fixator type for Pilon fractures (Kim H. J. et al., 2011; Kim SH. et al., 2011), primarily due to elevated stress concentrations in unstable fracture configurations. Previous biomechanical studies have established critical stress thresholds for complication-free healing. Stresses within the external fixator ranging from 436 to 750 MPa can lead to adverse outcomes (Morales et al., 2017), while the stress at the bone-pin interface must remain below 60 MPa to prevent pin loosening and re-injury at the pin tract (Morales et al., 2017). Supporting this, Della Rosa et al. (2022) reported a maximum stress of 50 MPa at the bone interface in a FE model of an aluminum external fixator. Furthermore, the ultimate tensile strength of cortical bone is approximately 193 MPa (Turner et al., 2001). Therefore, to mitigate complications, bone stress should not exceed 193 MPa, and pin tract stress should remain below 60 MPa.

In our study, only the Ilizarov external fixator applied to normal bone exhibited pin tract stresses exceeding the 60 MPa safety threshold during both gait phases (118.78 MPa in swing vs. 156.10 MPa instance). This indicates a potential risk of bone resorption or failure at the proximal tibial pin sites in normal bone. Notably, pin tract stresses remained below this critical level in osteoporotic bone for all fixator types. However, it is important to highlight that the highest stress in the Ilizarov group was observed on the tibiotalar articular surface of fragment 1 (the medial distal tibial fragment), reaching 67.40 MPa during the stance phase. This stress concentration is likely attributable to ankle joint dorsiflexion and talar extrusion against the anterior tibial plafond during weight-bearing. Such elevated stress not only risks re-injury to fragment 1 but may also jeopardize the stability of adjacent fragments. Additionally, the cartilage stress on the tibiotalar articular surface for all fixators far exceeded the reported poroelastic fracture toughness of 1.83 MPa (Stok and Oloyede, 2007), suggesting a potential risk for post-traumatic cartilage damage, joint pain, and late-stage arthritis.

Regarding fracture stability, optimal secondary bone healing requires controlled micromovement at the fracture site, ideally within 0.15–0.40 mm (Claes et al., 1995). While micromovement exceeding 2 mm during activity is associated with fixation failure and non-union (Ramlee et al., 2014), appropriate fixation can promote healing (Ramlee et al., 2020). Our analysis of articular surface stress revealed that the Ilizarov fixator generated the highest tibiotalar joint stress in both bone types across both gait phases, indicating a greater potential for joint surface damage. In contrast, the tibiotalar articular surface stress remained below 1.83 MPa in osteoporotic models fixed with both the Delta and Unilateral configurations during stance. During the swing phase, the Delta fixator in OP bone resulted in a joint surface stress of 1.87 MPa, which was the lowest among all configurations, highlighting its advantage in minimizing joint loading.

Furthermore, our results demonstrated that fracture fragment micromovement was consistently greater in osteoporotic bone than in normal bone under identical fixation and loading conditions, underscoring the necessity for more stable fixator constructs in OP patients. During the swing phase, all three fixators maintained fragment micromotion below the 2 mm failure threshold (Table 5). However, only the Delta external fixator maintained micromovement within the ideal 0.15–0.40 mm range for both bone types. The other two fixators resulted in micromovements between 0.40 mm and 2.00 mm. The maximum micromovement at the tibiotalar articular surface typically occurred in fragment 4 (anterolateral), except in normal bone fixed with the Delta fixator, where it was localized to fragment 3 (posterolateral). In osteoporotic bone model, the Delta fixator also showed the highest micromovement in fragment 4. Additionally, von Mises stress was more concentrated on the lateral distal tibial fragment (fragment 1) in the Unilateral and Ilizarov groups, but not in the Delta group. This differential stress and micromovement distribution suggests that the more elastic fixation provided by the Delta configuration may be more conducive to secondary bone healing during the swing phase. During the stance phase, fracture fragments fixed with the Delta external fixator demonstrated smaller and more stable micromotions (<2 mm) than those stabilized with the other two fixators in two bone conditons models. In contrast, fragments fixed with the Unilateral and Ilizarov fixators exhibited micromotions exceeding 2 mm, indicating potential fixation failure. Although the Delta fixator yielded the smallest fragment micromovement in normal bone during stance, the values still exceeded the ideal 0.40 mm threshold (Table 6). This suggests that none of the three fixator constructs may provide an ideal environment for fracture healing during full weight-bearing. It is noteworthy that fragment micromovement was consistently greater in osteoporotic bone than in normal bone for all fixators and across both gait phases (Tables 5, 6), implying that bone deterioration makes Pilon fractures more challenging to stabilize effectively.

Meanwhile, under identical bone quality and loading conditions, the Delta external fixator demonstrated the lowest fracture fragment stress and micromovement. It also induced the smallest stress and micromovement at the articular surface, resulting in the most stable and advantageous fixation overall. The similar micromovement observed in fragments 4 and 5 is likely due to stress concentration in the anterior distal tibia during gait cycles—especially in osteoporotic bone with reduced structural integrity. The superior stability offered by the Delta fixator may be attributed to its configuration, particularly the inclusion of a K-wire in the first metatarsal.

The first metatarsal K-wire, anchored to the posterior and lateral short rods of the Delta fixator, creates a triangular force vector that counteracts anterior shear forces on these fragments. As shown in Table 8 and Figure 6, this results in a 76%–87% reduction in anterior fragment micromovement compared to the other fixators: in osteoporotic bone during stance phase, Delta fixator limits Fragment 4 micromovement to 2.15 mm, whereas Unilateral and Ilizarov fixators allow 9.65 mm and 17.08 mm, respectively. Also this K-wire helps Stress Distribution Optimization. It transfers part of the load from the distal tibia to the forefoot, reducing stress concentration on the lateral distal tibial fragment (Fragment 1). The results (Tables 5, 6) demonstrate that the Delta fixator avoids excessive stress on Fragment 1 (Max_VMS = 9.44 MPa in OP stance phase), whereas the Unilateral (9.95 MPa) and Ilizarov (13.59 MPa) fixators exhibit higher stress concentrations in this region—mitigating the risk of fragment re-fracture or nonunion. Unlike the Ilizarov fixator (which relies on multiple rings for rigidity but lacks direct fragment fixation) or the Unilateral fixator (which provides linear stabilization only), the first metatarsal K-wire adds a third stabilization axis. This creates a rigid triangular framework that restricts translational and rotational micromovement (Figure 3b) while maintaining minimal elastic deformation—critical for secondary bone healing (optimal micromovement: 0.15–0.40 mm (Claes et al., 1995)). Our FEA confirms this balance: Delta fixator achieves swing-phase micromovement of 0.47 mm (OP bone) within the optimal range, whereas the other fixators exceed this threshold (Unilateral: 2.19 mm; Ilizarov: 3.93 mm). These mechanisms collectively explain why the Delta fixator outperforms the other configurations in maintaining biomechanical stability—directly attributable to the first metatarsal K-wire’s unique force-transmission and fragment-restriction effects.

While a key advantage of the Ilizarov system is its capacity to enhance rigidity in osteoporotic bone by adding more K-wires (Beris et al., 2010), the specific configuration used in this study, which lacked direct fragment fixation, proved to be the least stable. Conversely, the Delta fixator also allows for the addition of multiple K-wires in the distal tibia to augment stability, a flexibility not afforded by the Unilateral fixator.

We acknowledge that our FEA model does not include clinical factors such as soft tissue swelling, pin tract infection risk, or patient compliance—all of which influence real-world outcomes. To address this, we have expanded the discussion to contextualize our biomechanical findings with these factors: (1) Pin Tract Infection: While the Delta fixator uses two additional K-wires (compared to the Unilateral fixator), our FEA shows it maintains bone-pin interface stress below 60 MPa (the safety threshold for pin loosening (Morales et al., 2017)) in osteoporotic bone (Max_VMS = 9.44 MPa). This reduces the risk of pin tract infection (linked to excessive stress and loosening (Beumer et al., 2003)), offsetting the theoretical risk of additional pins. (2) Soft Tissue Compromise: The Delta fixator’s modular short rods and triangular design allow for greater adjustability to accommodate soft tissue swelling—critical in high-energy Pilon fractures with severe soft tissue injury. Unlike the rigid Ilizarov ring fixator (which may compress swollen soft tissues), the Delta fixator’s open configuration minimizes soft tissue irritation. (3) Patient Compliance: Early ambulation enabled by the Delta fixator’s stability improves patient compliance, as elderly patients are more likely to adhere to rehabilitation when experiencing less pain and better mobility.

In addition, Delta fixator may be more suitable for frail OP patients (elderly OP patients with Pilon fractures often frail) because of its stability. Our model’s focus on “early limited weight-bearing” (consistent with clinical guidelines for this population (Nozaka et al., 2020; Rolvien and Amling, 2022)) aligns with their functional status: partial weight-bearing and not full ambulation. The Delta fixator’s triangular configuration and first metatarsal K-wire minimize fracture fragment micromovement (1.78 mm in OP bone during stance phase), reducing pain and improving balance—critical for frail patients at high risk of falls. Unlike the rigid Ilizarov fixator (which may restrict ankle motion) or the unstable Unilateral fixator (which requires more patient effort to maintain balance), the Delta fixator balances stability and flexibility. This allows frail patients to perform seated ankle exercises and short-distance ambulation with assistive devices, mitigating disuse osteoporosis and muscle atrophy (Rolvien and Amling, 2022). Moreover, the Delta fixator’s reduced bone-pin interface stress (<60 MPa in OP bone) decreases the risk of pin loosening and infection—complications that disproportionately impact frail patients with compromised wound healing.

In conclusion, our FE analysis showed that the bone stress in Pilon fractures fixed with any of the three external fixators did not exceed the ultimate tensile strength of bone (193 MPa) under either normal or osteoporotic conditions. During the loading simulations over two gait cycles, the Delta external fixator was identified as the most suitable and stable construct among the three fixation devices, particularly in the osteoporotic Pilon fracture. In clinical practice, additional K-wires are required to stabilize and anchor fracture fragments to the external fixator. Notably, the Delta and Ilizarov external fixators offer more ample and favorable anchoring space compared with the unilateral external fixator, which lacks sufficient anchoring points to accommodate the additional K-wires needed for the comprehensive stabilization of all fracture fragments. Based on its superior mechanical performance, the analysis results may inform a prioritized surgical approach for the management of Pilon fractures in specific high-risk patient cohorts (i.e., frail OP patients and ORIF-ineligible patients).

This construct affords a more favorable biomechanical environment to facilitate early exercise, ambulation, and limited weight-bearing in these patients. While clinicians should advise patients to limit prolonged standing to mitigate the potential risk of fixation failure, our finite element analysis (FEA) results offer a robust rationale for the clinical application of the Delta fixator in osteoporotic Pilon fractures to inform future clinical trials.

## 5 Limitations

This study has several limitations:

1. Although the material properties used in this model are consistent with those in the literature (Ramlee et al., 2020) and the results are reasonably reliable, they still differ from real human bone tissue. Further experimental validation is required to confirm the stability of these configurations.
2. This study established a single Ruedi-Allgower type III fracture model. Other strategies, such as limited internal fixation combined with external fixation or ORIF alone, have also demonstrated good clinical efficacy and mechanical stability for this fracture type. Our study lacks a direct comparison with internal fixation methods and a wider array of external fixator configurations.
3. The model did not incorporate the effects of surrounding muscles. Because muscle components have not been appropriately addressed yet (Morales et al., 2017). Moreover the soft tissue materials of patients with osteoporosis, sarcopenia, fracture and swelling could not be evaluated too. After the accurate soft tissue materials have been measured, future analyses should include muscle forces, with particular consideration given to the muscle status of elderly OP patients.
4. The initial model adopted a constant friction coefficient (0.4) for the bone-pin interface to isolate the intrinsic biomechanical differences between fixator configurations. This simplification allowed us to establish a baseline comparison of fixator design performance, independent of time-dependent tissue changes. However, we acknowledge that bone-pin interaction evolves throughout healing, and this dynamic process must be integrated to strengthen clinical relevance. Bone-pin interface properties are time-dependent changes. With the time goes, fracture healing make fixator more reliable. And there was more significant to simulate and analyze the fracture initial stage. It may be more persuasive for the conclusion. In future analyses, we would like to simulate and analyze the Pilon fracture’s late phase with the Delta fixation.
5. There is a lack of standardized quantitative data on the average magnitude of cortical thinning or cancellous bone widening in OP patients with distal tibial fractures. Future studies will integrate clinical CT data from a cohort of OP Pilon fracture patients to quantify individual-specific cortical thickness reductions, enabling more anatomically realistic FE modeling and further validating the Delta fixator’s performance in geometrically accurate OP bone constructs.

## Data Availability

The original contributions presented in the study are included in the article/Supplementary Material, further inquiries can be directed to the corresponding authors.
